# Rodent control to fight plague: field assessment of methods based on rat density reduction

**DOI:** 10.1111/1749-4877.12529

**Published:** 2021-03-30

**Authors:** Soanandrasana RAHELINIRINA, Kathryn SCOBIE, Beza RAMASINDRAZANA, Voahangy ANDRIANAIVOARIMANANA, Fanohinjanaharinirina RASOAMALALA, Lovasoa Nomena RANDRIANTSEHENO, Jerry Sylvio RAKOTONIAINA, Olivier GORGÉ, Xavier LAMBIN, Eric VALADE, Sandra TELFER, Minoarisoa RAJERISON

**Affiliations:** ^1^ Institut Pasteur de Madagascar Plague Unit Antananarivo Madagascar; ^2^ School of Biological Sciences University of Aberdeen Aberdeen UK; ^3^ Central Laboratory for Plague Ministry of Public Health Antananarivo Madagascar; ^4^ Institut de Recherche Biomédicale des Armées Paris France

**Keywords:** fleas, Madagascar, plague, rodents, surveillance

## Abstract

Rodents represent a serious threat to food security and public health. The extent to which rodent control can mitigate the risk from rodent‐borne disease depends on both the effectiveness of control in reducing rodent abundance and the impact on disease epidemiology. Focusing on a plague‐endemic region of Madagascar, this study compared the effectiveness of 3 methods: live‐traps, snap‐traps, and rodenticides. Control interventions were implemented inside houses between May and October 2019. Tracking tiles monitored rodent abundance. Rodent fleas, the vector involved in plague transmission, were collected. Rodent populations consisted of *Rattus rattus* and *Mus musculus*. In terms of trap success, we found that our live‐trap regime was more effective than snap‐traps. While all 3 control strategies appeared to reduce in‐house rodent activity in the short term, we found no evidence of a longer‐term effect, with in‐house rodent abundance in treated sites comparable to non‐treatment sites by the following month. Endemic flea, *Synopsyllus fonquerniei*, is a key plague vector usually found on rats living outdoors. Although we found no evidence that its abundance inside houses increased following control, this may have been due to a lack of power caused by significant variation in *S. fonquerniei* abundance. The presence of *S. fonquerniei* in houses was more likely when *S. fonquerniei* abundance on outdoor rats was higher, which in turn correlated with high rat abundance. Our results emphasize that control strategies need to consider this connectivity between in‐house rat–flea populations and the outdoor populations, and any potential consequences for plague transmission.

## Graphical abstract

Focusing on a plague‐endemic region of Madagascar, this study compared the effectiveness of 3 methods: live‐traps, kill‐traps, and Kartman bait station containing anticoagulant rodenticide. Live‐trap regime was more effective than kill‐traps. Wire‐mesh BTS trap catch a higher number of *Rattus rattus*, the main reservoir of plague in Madagascar. Control strategies need to consider the connectivity between in‐house and outdoor rat–flea populations and any potential consequences for plague transmission.

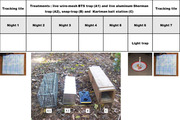

## INTRODUCTION

Rodents pose a serious threat to agricultural productivity and public health. Across the globe, pre‐ and post‐harvest losses to rodent pests contribute to malnutrition and global food insecurity (Meerburg *et al*. 2009a). Rodents are also reservoirs for various zoonotic diseases transmissible to humans and animals (Meerburg *et al*. 2009b). Thus, the effective control of rodents could have significant impacts on health and well‐being. However, due to their behavioral plasticity, life history traits, and high breeding potential, the control of rodents is notoriously difficult (Capizzi *et al*. 2014).

In ecologically based rodent management (EBRM), control programs are designed around the target species’ population dynamics as well as the local ecological and sociocultural setting (Singleton *et al*. 1999). Rodent pests often occupy multiple habitats within an area, and so key to this approach is understanding their spatial and seasonal population dynamics, in particular the density‐ and resource‐dependence of movement and reproduction, and using this understanding to inform decisions on where and when to target control. So far, studies have focused on the application of EBRM within agricultural systems, and localized, targeted control has proved effective at significantly reducing rodent damage on smallholder farms (Belmain *et al*. 2003; Singleton *et al*. 2005; Brown *et al*. 2006; Palis *et al*. 2011). However, targeted control strategies could impact on the epidemiology of, and risk from, rodent‐borne zoonoses.

In some circumstances, control may increase disease prevalence in reservoirs through impacts on movement and contact rates, as observed for bovine tuberculosis (bTB) in badgers (Bielby *et al*. 2016; Smith & Delahay 2018) and leptospirosis in urban populations of brown rats (Lee *et al*. 2018). Rodent populations are also able to compensate for removal through immigration from surrounding areas (Krebs *et al*. 1978; Sullivan *et al*. 2001, 2003), as well as through increased reproduction and earlier maturation (Singleton *et al*. 1999). Thus, although reducing rodent abundance could play a role in reducing the burden of rodent‐borne disease, control programs must carefully consider local epidemiological cycles of rodent‐borne zoonoses, as well as the rodent population dynamics.

Plague is one of the most infamous of rodent‐borne zoonoses. Madagascar is one of the countries with the highest plague burden in the world (Bertherat 2019). In 2017, the country experienced a particularly severe outbreak, with over 2400 cases notified in only 4 months (Randremanana *et al*. 2019). In most years, the majority of cases are reported from rural areas in the Central Highlands (at altitudes >800 m) between September and March (Migliani *et al*. 2006). Most human plague cases are bubonic after infection with *Yersinia pestis* (Lehmann & Neumann, 1896) van Loghem, 1944, the causative agent, via the bite of infected rodent fleas. In rural areas of the Central Highlands, the black rat *Rattus rattus* (Linnaeus, 1758) is the main reservoir while 2 flea vectors have been implicated: *Xenopsylla cheopis* (Rothschild, 1903) the cosmopolitan tropical rat flea, and *Synopsyllus fonquerniei* (Wagner & Roubaud, 1932) an endemic flea which is restricted to altitudes >800 m (Andrianaivoarimanana *et al*. 2013).

Madagascar's public health service has developed strong systems to monitor plague incidence, but despite the zoonotic nature of the pathogen, animal‐based plague surveillance is not routinely conducted and most surveillance and control efforts focus on managing outbreaks as opposed to prevention. These efforts have occasionally used live‐traps to remove rodents (Rasoamanana *et al*. 1998), while the use of rodenticide or snap‐traps has generally been discouraged due to the risks posed by fleas leaving dead hosts. Locally made wooden tunnel Kartman bait stations (KBS) (Kartman 1958) containing both insecticide and anticoagulant rodenticide have been shown to be effective to control rats in an urban Malagasy plague focus (Ratovonjato *et al*. 2003), but subsequent trials in a rural focus recorded disappointing results in terms of flea control (Miarinjara *et al*. 2019).

Following the 2017 outbreak, there has been renewed interest in understanding how control which targets rodent and flea populations could play a role in reducing the risk from plague (Vallès *et al*. 2020). One option being discussed in Madagascar is targeting in‐house rat populations. As highlighted above, the impact of such localised control on both rodent dynamics and disease epidemiology needs to be considered.

Most of Madagascar's Central Highlands are a mosaic of villages, agricultural fields, fallow land, and pasture. *Rattus rattus* is found throughout these habitats, with in‐house populations generally understood to be distinct from those found outside, as evidenced by the variation in flea species parasitizing in‐house and outdoor rats; while *X. cheopis* is found predominantly on *R. rattus* living indoors, *S. fonquerniei* is typically found on *R. rattus* captured outdoors (Duplantier *et al*. 2005; Andrianaivoarimanana *et al*. 2013). Ecological studies in a limited number of locations indicate that *S. fonquerniei* shows a seasonal cycle, with numbers peaking at the end of the cool, dry period (September–November) (Klein 1966). It is at this time that rats are more likely to move the short distances into houses from surrounding areas, driven by the availability of resources (Rahelinirina *et al*. 2010). These seasonal changes coincide with the start of the human plague season (Migliani *et al*. 2006). It is therefore essential that rat control programs in these heterogeneous landscapes take into account the impact of rat movements on both population recovery and disease risk.

With a view to informing how rodent control could contribute to future plague prevention strategies in Madagascar, we conducted a study in villages within the plague‐endemic area to test the efficacy of 3 different types of control of in‐house rodents: live‐traps, snap‐traps, and KBS containing rodenticide and insecticide. To minimize any risks associated with the lethal control of rodents in the absence of prior flea control, the study was carried out during the low plague season (May‐September) in an area without any reported plague cases during the preceding 5 years. The specific objectives of the project were to (i) compare the effectiveness of live‐traps and snap‐traps for capturing rodents in houses; (ii) evaluate the effect of treatments on in‐house rodent abundance in the short‐term (over 5 consecutive nights) and long‐term (over 5 consecutive months); (iii) assess evidence of increased reproduction in in‐house rodent populations subjected to control; (iv) assess the impact of treatments on fleas inside houses, including the abundance of *X. cheopis* (the species mostly found on rodents in houses) and the presence of *S. fonquerniei* (the species typically found on rodents outside); and (v) assess how in‐house rodent and flea populations are impacted by the abundance of rodents and *S. fonquerniei* in the immediate surrounding areas.

## MATERIALS AND METHODS

### Study sites

**Figure 1:**
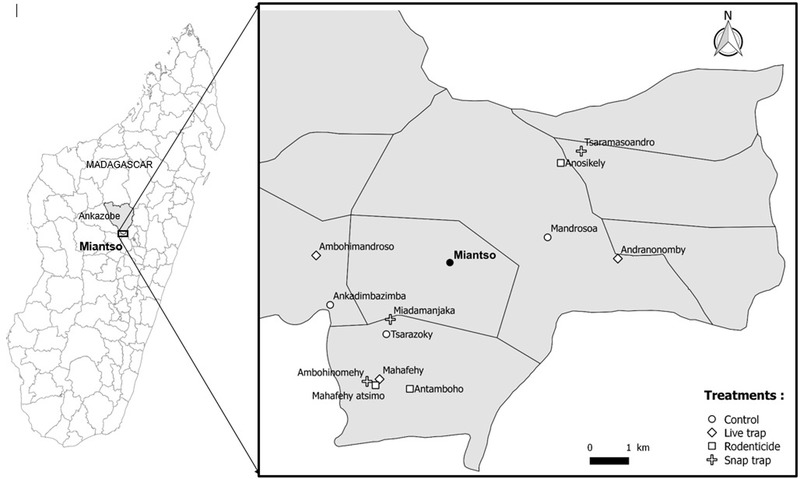
Location maps of the study sites: zoom into the Miantso district. The symbols represent the treatment groups assigned to each study site between May and September 2019.

The study was conducted in the commune of Miantso (–18.718289°S; 47.148315°E), in the district of Ankazobe, a plague endemic region of Madagascar. The location is shown in Fig. 1. Ankazobe is located on Madagascar's High Plateau, averaging 1400 m above sea level, and is characterized by vast stretches of grassland interspersed with agricultural fields and paddy fields for growing rice, scrub land, and small patches of forest. The region experiences a dry‐cold season (May to September) and wet‐warm season (October to April).

Twelve (12) villages were selected based on their size (between 9 and 22 houses) and accessibility. Most families practiced subsistence agriculture, and houses were traditionally constructed with mud walls and grass‐thatched roofs. For security, domestic animals and food crops are often kept within the household. The field study was carried out between May and October 2019, during the dry‐cold season which is associated with low prevalence of plague. To ensure consistency between sites, control treatment methods were implemented monthly by the project team. In each village, we obtained verbal consent from the local leader and each household head to carry out activities. Mean household participation was 88.1%, ranging from 60% to 100%.

### Rodent control treatment

**Figure 2:**
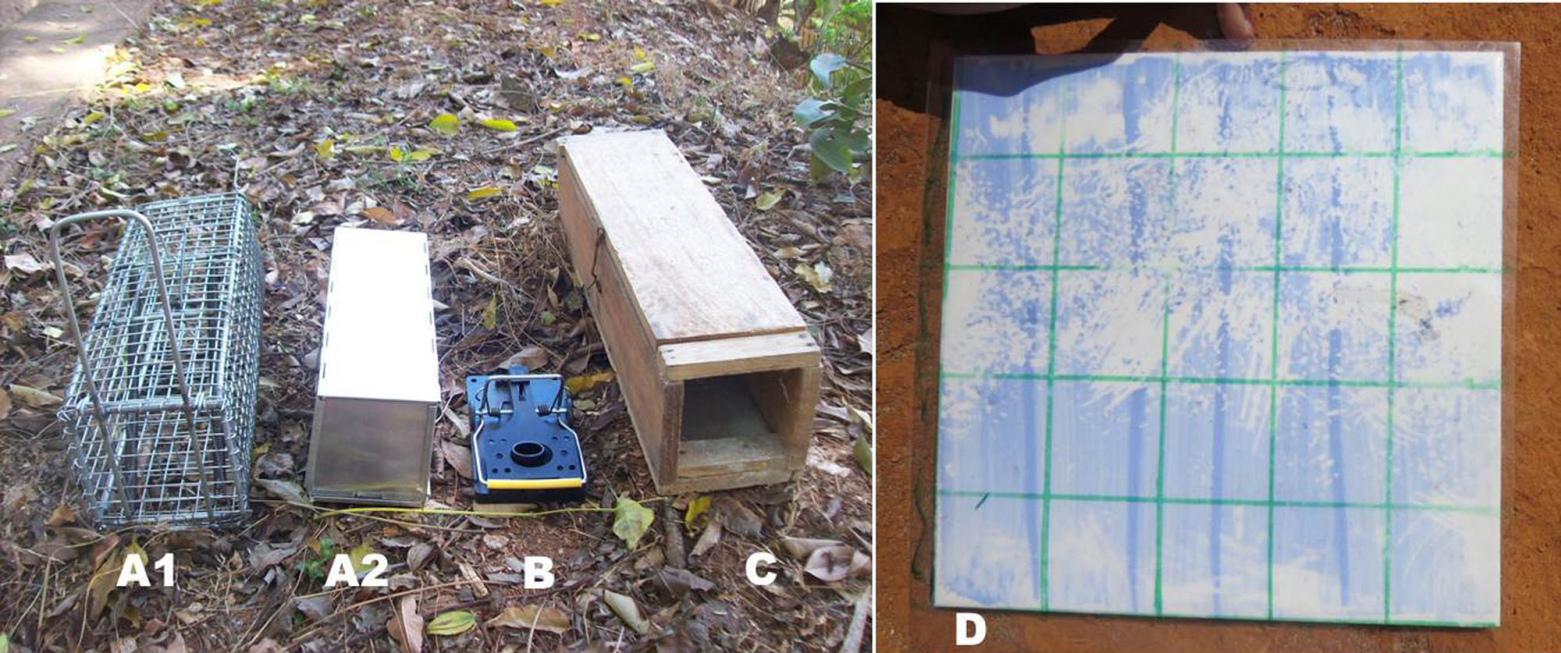
Control treatments: live wire‐mesh BTS trap (A1) and live aluminum Sherman trap (A2), snap‐trap (B), and Kartman bait station (C) (photo: SR). Rodent tracking tile (D): white ceramic tile painted with a mixture of blue chalk powder, white spirit and motor oil pictured with acetate sheet divided into a 5 × 5 grid. In this example, the tile was given a tile activity score (TAS) of 25 (photo: KS).

This experiment tested the effectiveness of 3 different types of rodent control treatments: live‐traps, snap‐traps, and rodenticide delivered in KBS (Fig. 2). Three villages were randomly assigned to each of these treatments, with all participating households within a village implementing the same treatment. For comparison, the study included a control group of 3 villages, which did not implement any rodent control treatment, herein referred to as the non‐treatment group.

In villages within the live‐trap treatment, each household was provided with 2 wire‐mesh BTS traps (30L × 10W × 10H cm) and one aluminum Sherman trap (23L × 7.5W × 9H cm). In the snap‐trap group of villages, each household received 3 snap‐traps (ABS plastic and steel, 14L × 7.5W × 6.5H cm). Traps were baited with peanut butter. In the villages within the KBS treatment, each household received 2 KBS (40L × 10W × 10H cm) containing 150 g of insecticide (Fenitrothion 2%) powder (Prochimad, Madagascar) and anticoagulant rodenticide (chlorophacinone 0.05%) bait block (Koderat, BHL, Madagascar), a lethal dose of which will kill a rat 4‐days post‐ingestion. Traps and KBS were set at fixed locations inside houses, in areas thought to be used by rats but out of reach of children and non‐target species. Control treatments were left in place for 5 consecutive nights, every 4 weeks, between May and September 2019 (amounting to a total of 5 treatment sessions). Three villages within the non‐treatment group did not receive any rodent control.

Captured animals were collected every morning and traps were reset at the same place. In households using snap‐traps, residents were permitted, but not required, to immediately remove rodent carcasses to avoid the risk of dispersal of rodent fleas. They were provided with zip‐loc bags and gloves to handle and store dead rats.

In October 2019, 6 months after initiating the control trial and one month after the last treatment cycle, we conducted a post‐treatment trapping session in all 12 villages to evaluate the long‐term impact of the three treatments on rodent abundance. In each house, one BTS and one Sherman trap were installed for 3 nights and were baited with peanut butter. Over the same 3 nights, to assess rodent abundance in the surrounding areas, we installed 40 BTS traps outside of houses in each of the 12 villages. These outdoor traps were positioned within a 3–80 m radius of houses, both within the village periphery and in surrounding agricultural areas, in areas thought to be used by rats (e.g. alongside walls or in covered areas of vegetation) and at intervals of ≥ 20 m.

Captured rodents were euthanized and identified to species level. Rodent handling was done in accordance with directive 2010/63/EU of the European Parliament (http://eur‐lex.europa.eu/Lex‐UriServ/LexUriServ.do?uri = OJ:L:2010:276:0033:0079:EN:PDF) and the guidelines of the American Society of Mammologists for the use of wild animals in research and education (Sikes *et al*. 2016). Animals were sexed, weighed, and measured. Fleas were removed from the mammals over a wash basin, stored in 95% alcohol, and identified. Blood samples were collected on seropads. Spleen samples were preserved in Cary‐Blair transport medium.

### Tracking tiles

Tracking tiles measure rodent activity by recording their footprints and other distinguishable rodent marks (i.e. scratches and tail swipes), and were used to provide a proxy measure of rodent abundance both inside and outside houses in all twelve villages. White ceramic tiles (20 × 20 cm) were painted with a mixture of 32.0% blue chalk powder, 64.0% white spirit, and 4.0% motor oil (an adaptation of the method trialled by Whisson *et al*. 2005) and allowed to dry (Fig. 2D). Three tiles were set in each house involved in the trial for one night before trap or KBS installation and one night after their removal (for schedule of activities see Table S1, Supporting Information). Tiles were simultaneously set in‐houses within the non‐treatment group. Outdoors, 20–25 tiles were baited with one teaspoon (4.0 g) of dry rice and installed within a 3–80 m radius of houses and at intervals of ≥ 20 m and left in position for one night. Tiles were positioned at the same place as traps and KBS or, in the case of non‐treated households and outdoor tiles, in areas known or thought to be used by rats.

The following morning, a 20 × 20 cm acetate sheet divided into a 5 × 5 grid was placed over each tile, and the number of squares containing rodent marks was counted to assign each tile an activity score out of 25 (Fig. 2D). The process was repeated during all treatment sessions. As such, a tile activity score (TAS) out of 75 was obtained for each household (or out of 25 for the pre‐treatment visit, during which one tile was set per house) immediately before (TASHH_pre_) and after (TASHH_post_) each control treatment session. For outdoor areas, each tile obtained a score (TASEXT) out of 25 immediately before each control treatment session, and during the post‐treatment session.

### Diagnostic tests

Identification of *Y. pestis* infection in captured small mammals was performed using a molecular test followed by culture if positive. DNA from spleen sample was extracted using QIAamp DNA kit according to the manufacturer's instruction. PCR screening was done according to the new diagnosis algorithm of the Central Laboratory for Plague based on real time and conventional PCR (Janse *et al*. 2013; D'ortenzio *et al*. 2018; Randremanana *et al*. 2019). For PCR positive samples, a bacteriological culture using Cefsulodin Irgasan Novobiocin (CIN) selective medium for *Y. pestis* was used (Rasoamanana *et al*. 1996).

Detection of anti‐F1 IgG antibodies in blood samples was conducted by enzyme‐linked immunosorbent assay (ELISA) as previously described (Andrianaivoarimanana *et al*. 2012). For the revelation step, an anti‐rat IgG peroxidase conjugate (Sigma, 1:15000), an anti‐mouse IgG peroxidase conjugate (Sigma, 1:1500) were used for rodents and protein A peroxidase conjugate (Sigma, 1:5000) for shrews.

### Collection of domestic fleas

To monitor the presence of fleas inside houses, a light trap was used to collect free fleas on the final night of treatment (or the equivalent night, at non‐treatment villages). Each household was provided with one light trap (a shallow dish containing soapy water, with a candle placed at the center), which was positioned in the center of the main living room. The candle was lit at dusk and extinguished the following morning. Fleas are attracted to the light and fall into the soapy water (Kilonzo 1977). These were collected using fine forceps and preserved in 95% ethanol for identification under binocular microscope.

### Data analysis

Analysis was conducted in R (R Core Team 2020). We first outline our general approach to data analysis, before the details for specific analyses. Most of the responses of relevance to our objectives involved count data. Therefore, where Generalized Linear Models (GLM) or Generalized Linear Mixed Models (GLMM) were used, we first identified the most appropriate distribution for each analysis. GLM(M)s were constructed using the package glmmTMB (Brooks *et al*. 2017), and we used Akaike's information criterion (AIC) to compare Poisson, negative binomial (NB), zero‐inflated Poisson (ZIP), and zero‐inflated negative binomial (ZINB) models (Table S2, Supporting Information). ZIP and ZINB models account for an excess of zero counts and assume that the excess zeros are generated by a separate process and can be modeled independently. Thus, these models have 2 parts, a count model fit to a Poisson or NB distribution and a logit model for excess zeros. Here, an example of a process that could result in an excess of zeros could be the true absence of rodents from a house. Model comparisons were made using the full model, which included any fixed and random effects hypothesized to be important (details below for each analysis). Subsequently, a backward selection approach based on likelihood ratio tests (LRT) was used to identify significant fixed effects, using a *P*‐value threshold of *P* <0.05. For ZIP or ZINB models, selection was conducted separately for the zero and count parts of the model. Full models and the best‐selected models for each analysis are given in Table S3, Supporting Information.

We first explored natural variation of rodent abundance between villages, inside and outside houses. As we do not have data on background rodent density available from the site, we used TASHH_pre_ and TASEXT for May, respectively, as the response variables, and checked for any systematic variation in pre‐treatment abundance between treatment groups. For TASEXT, data were left‐skewed and so the number of squares without rodent marks (empty squares) per tile was used as the response variable (TASEXT_empty_). We ran GLMs with village as a fixed effect, as well as GLMMs with treatment as a fixed effect and village as a random effect. A ZINB model provided the best fit to the in‐house rodent abundance index data (TASHH_pre_) and a NB model fitted the outdoor rodent abundance index data (TASEXT_empty_) (Table S2).

To compare the efficiency of the 2 different trap regimes for catching rodents (snap‐traps vs live‐traps), we used GLMMs to model capture rates using data from the 6 villages and all 5 treatment sessions. Analyses were limited to households with evidence of rodent infestation (houses with tile activity score >0 and/or where rodent captures were ≥1). Three analyses were conducted: all rodent captures, rat‐only captures, and mouse‐only captures. NB models provided the best fit to all 3 datasets (Table S2, Supporting Information). Variation in capture rates could have been due to variation in rodent abundance or seasonal differences in behavior. We therefore included TASHH_pre_ and TASEXT as proxies for rodent abundance (where TASHH_pre_ was calculated for each house and treatment session as the number of marked squares divided by the number of readable tiles to give a TAS out of 25, and the average TASEXT was calculated for each village and treatment mission), and a variable to describe temporal variation. The full model also included interactions between trap regime and TASHH_pre_, and between trap regime and the variable describing temporal variation. In species‐specific analyses we included the interaction of trap regime and the presence of the other rodent species (PR_RR_ or PR_MM_) in the house to test for evidence of inter‐specific competition for traps. Prior to final selection of the full model, we used AIC to select the best representation of temporal variation, comparing 3 different ways of modeling the temporal variation: VISIT_cat_, where visit number was a 5‐level categorical variable; SEAS, a 3‐level categorical variable with visit 1, visits 2/3 and visits 4/5 as levels, and VISIT_cont_ which allowed for a linear change over time. Models included house nested within village as random effects, and the natural log of the number of available traps as an offset. Corrected trap nights were calculated as the number of traps containing rodents or not sprung plus half the number of traps which were sprung or had bait removed but which had not caught a rodent (Theuerkauf *et al*. 2011). This calculation apportions a trap availability score of 0.5 to traps which either had sprung but failed to catch a rodent or which had failed to spring when the bait was removed.

To evaluate the short‐term effect of treatment on in‐house rodent abundance, we used paired Wilcoxon tests to compare TASHH_pre_ and TASHH_post_, with a separate analysis for each treatment. Households where TASHH_pre_ was equal to zero were excluded from the analysis, thus excluding those with no evidence of rodent infestation prior to treatment.

To explore long‐term effects of treatment, we tested for variation between treatment groups in the post‐treatment abundance of rodents inside houses, using rodent capture data from the post‐treatment trapping session. Three analyses were conducted: all rodent captures, rat‐only captures, and mouse‐only captures. We used GLMMs with treatment as a fixed effect, natural log of the number of available traps as an offset and village as a random effect. To further explore spatial variation, we ran GLMs with village as a fixed effect. Poisson models provided the best fit to the rat capture data and negative binomial to the mouse and combined rodent capture data (Table S2, Supporting Information).

In addition, we explored changes in the in‐house abundance index over the course of the experiment and evaluated differences in this between treatment groups and villages, using GLMMs and TASHH_pre_ from all 5 treatment sessions. A ZINB model provided the best fit to the data (Table S3, Supporting Information). The full model included an interaction between treatment and temporal variation (modeled as described above). As the analysis included the first treatment session (before any impact of treatment), we expected this interaction to be significant if any of the treatments yielded long‐term effects persisting between treatment sessions. To determine whether in‐house rodent abundance was influenced by outdoor rodent abundance, the full model also included TASEXT. House nested within village were included as random effects and the natural log of the number of tile squares as an offset.

To assess evidence of increased reproduction in in‐house rat populations subjected to control, we used data from the post‐treatment trapping session and compared the population structure and gestation rate between treatment and non‐treatment sites. Due to low sample size we used Fisher tests. For population structure, non‐pregnant rats were classified into 4 age classes based on weight (Females—class 1: <40 g, class 2: 40–79 g, class 3: 80–119 g, class 4: >120 g; Males—class 1: <45 g, class 2: 45–89 g, class 3: 90–134 g, class 4: >135 g). Gestation rates were based on females in age categories 2, 3, and 4.

To examine the impact of treatment on rodent flea abundance, we used the data from rodents caught in houses during the post‐treatment capture session. The flea burden of rats was compared across treatment groups using a Kruskal Wallis test. We assessed the 2 flea species separately as *X. cheopis* abundance could have been directly influenced by any effect of treatment on in‐house rodent abundance or, in the case of KBS treatment, by the use of insecticide, while *S. fonquerniei* abundance could have been influenced by any impact of treatment on the movement of outdoor rodents into houses. A Mann–Whitney *U*‐test was used to compare the *S. fonquerniei* index of rats caught outside at villages where *S. fonquerniei* were and were not collected from rats inside houses. For each village, the *S. fonquerniei* index of rats caught outside was calculated as the total number of *S. fonquerniei* divided by the number of rats caught.

To explore spatial and temporal variation in outdoor rodent abundance, we analyzed TASEXT_empty_ from May–October using a GLMM containing village, a variable to describe temporal variation (modeled as described above but with October as a separate level for categorical variables) and their interaction. A NB model provided the best fit to the data (Table S2, Supporting Information). Outdoor tiles were placed in the same location each month and given a unique number, which was included as a random effect. We also tested for variation between villages using a GLM and outdoor rat capture data from the post‐treatment trapping session, which was best fit to a Poisson distribution (Table S2, Supporting Information), with the natural log of the number of available traps included as an offset. To examine the relationship between the *S. fonquerniei* burden and rat abundance, GLMMs were constructed with the number of *S. fonquerniei* collected from each rat during the post‐treatment session as the response. To determine whether flea burden was associated with past or present rodent abundance, we assessed models that included either TASEXT from the post‐treatment session, TASEXT from the preceding month, or the average TASEXT from the preceding 3 months as fixed effects, fitted to a ZINB distribution (Table S3, Supporting Information). Village was included as a random effect.

## RESULTS

### Pre‐treatment rodent abundance

There was variation between villages in the probability of in‐house rodent infestation (i.e. the probability of recording a zero TASHH_pre_; χ^2^(10) = 26.7, *P* = 0.003) and in TASEXT_empty_ (χ^2^(11) = 161.6, *P* < 0.005) prior to the start of treatment. However, we detected no systematic variation between treatment groups in the probability of in‐house rodent infestation (χ^2^(3) = 5.7, *P* = 0.13), the abundance index in infested houses (χ^2^(6) = 6.0, *P* = 0.11), or outdoor rodent abundance (χ^2^(3) = 4.0, *P* = 0.26).

### Success of different trap types

**Figure 3:**
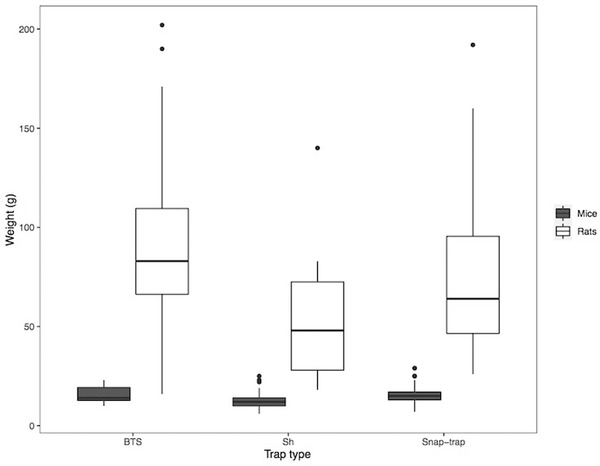
Median, and upper and lower quartiles, of weights (g) of *M. musculus* and *R. rattus* caught by wire‐mesh BTS, Sherman (Sh), and snap‐traps during treatment sessions.

During the treatment period, the 3 trap models used in the 2 trapping regimes captured 393 small mammals, belonging to 3 species: 170 in Sherman (162 *Mus musculus* Linnaeus, 1758 and 8 *R. rattus*) from an effort of 589 trap nights, 105 in BTS (9 *M. musculus* and 96 *R. rattus*) from an effort of 1159 trap nights, and 114 in snap‐traps (82 *M. musculus*, 31 *R. rattus*, and 1 *Suncus murinus* Linnaeus, 1758) from an effort of 2008 trap nights. A full breakdown of small mammal captures is provided in Table S4a, Supporting Information. The weight of captured animals varied between 16 and 202 g for *R. rattus* and 6 and 29 g for *M. musculus* (Fig. 3). There was no significant difference in the sex ratio of *R. rattus* captured from the 3 traps (BTS χ^2^(1) = 0.01, *P* = 0.919; Sherman χ^2^(1) = 0.5, *P* = 0.479; Snap χ^2^(1) = 1.6, *P* = 0.209).

**Figure 4:**
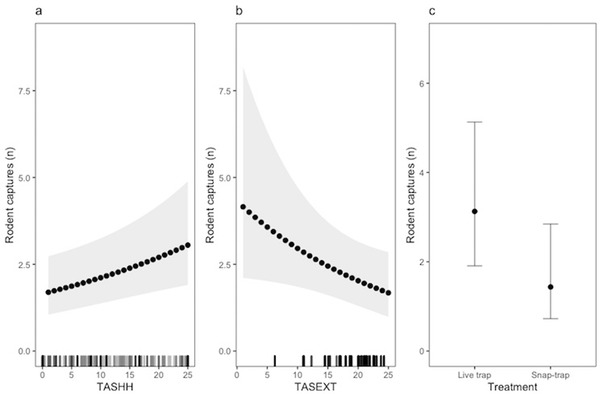
Predicted rodent captures and 95% confidence intervals derived from a negative binomial Generalized Linear Mixed model illustrating (a) the effect of in‐house tile activity score (TASHH) at the median outdoor tile activity score (TASEXT), (b) the effect of TASEXT at the median TASHH, and (c) the effect of trap type at median TASHH and TASEXT. Tick marks indicate the distribution of observations at each value of TASHH and TASEXT.

**Figure 5:**
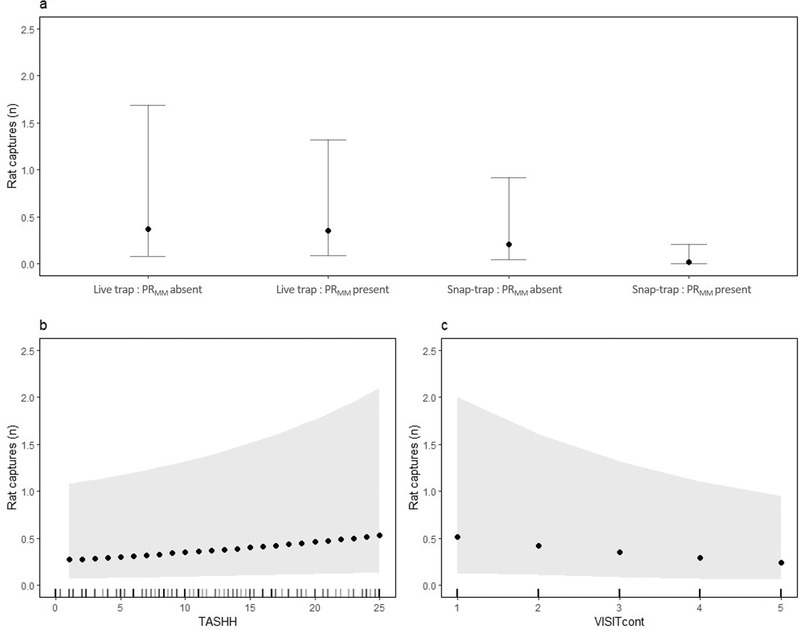
Predicted *R. rattus* captures and 95% confidence intervals derived from a negative binomial Generalized Linear Mixed model illustrating (a) the interaction effect of trap regime and presence of *M. musculus* (PR_MM_) at the median in‐house tile activity score (TASHH) and with visit (VISIT_cont_) fixed at 3, (b) the effect TASHH with VISIT_cont_ fixed at 3, and (c) the effect of VISIT_cont_ at the median TASHH. Tick marks indicate the distribution of observations at each value of TASHH and VISIT_cont_.

**Figure 6:**
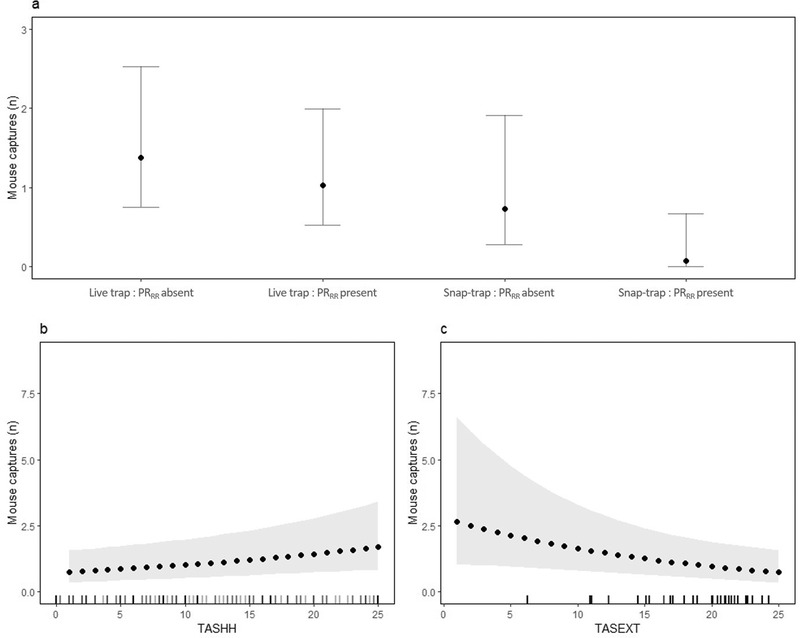
Predicted *M. musculus* captures and 95% confidence intervals derived from a negative binomial Generalized Linear Mixed model illustrating (a) the interaction effect of trap regime and presence of *R. rattus* (PR_RR_) at the median in‐house tile activity score (TASHH) and outdoor tile activity score (TASEXT), (b) the effect of TASHH at the median TASEXT, and (c) the effect of TASEXT at the median TASHH. Tick marks indicate the distribution of observations at each value of TASHH and TASEXT.

As expected, traps caught more animals when the house tracking tiles index (TASHH_pre_) indicated higher abundance (All rodents: χ^2^(1) = 7.73, *P* = 0.005, Fig. 4; Rats: χ^2^(1) = 3.0, *P* = 0.08, Fig. 5; Mice: TASHH_pre_ χ^2^(1) = 7.6, *P* = 0.006, Fig. 6). For rats, we also found evidence of a seasonal decrease in capture rate (χ^2^(1) = 5.0, *P* = 0.03, Fig. 5). Having accounted for these sources of variation in capture rates, we found strong evidence of a difference between the 2 trapping regimes with higher capture rates at the live‐trap sites. When all rodent captures were analyzed together, the effect of trap type was a straight additive effect (χ^2^(1) = 5.3, *P* = 0.02, Fig. 4). However, when the species were analyzed separately, we found the lower capture rate for snap‐traps was particularly apparent when the other species were present in the house (Rats: χ^2^(1) = 7.2, *P* = 0.007, Fig. 5; Mice: χ^2^(1) = 6.2, *P* = 0.01, Fig. 6). Interestingly, we found lower rodent capture rates when the outdoor tracking tiles index indicated higher abundance (χ^2^ (1) = 4.2, *P* = 0.04, Fig. 4), with this effect apparently driven by mice as there was no such relationship for rats alone but a strong effect for mice (χ^2^(1) = 5.5, *P* = 0.02, Fig. 6). Examination of the random effects indicated that, for rats, there was substantial variation in capture rates between houses within villages, while for mice most variation was between villages. Final models are presented in Table S5, Supporting Information.

### Short‐term effect of control

**Figure 7:**
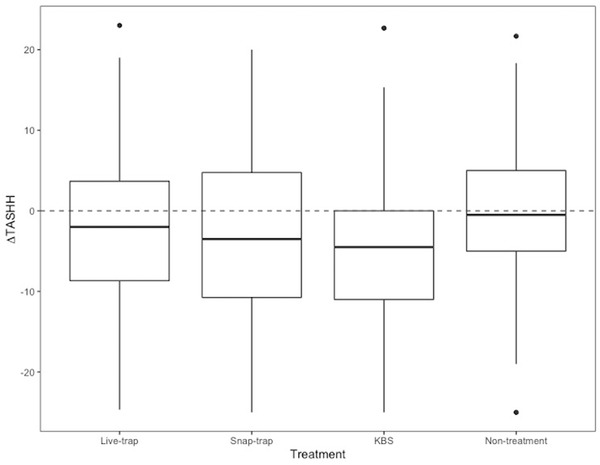
Median, and upper and lower quartiles, of changes in in‐house tile activity score (∆TASHH) between pre‐treatment (TASHH_pre_) and post‐treatment (TASHH_post_) for each house during each treatment session. Households are grouped by control treatment.

Excluding households with no evidence of rodent activity prior to treatment (i.e. excluding households where TASHH_pre_ = 0), the median change in in‐house tile activity score immediately after treatment (calculated for each house and treatment session as: TASHH_post_ − TASHH_pre_) was negative in households within the live‐trap (−2.00 [IQR −8.67 − 3.67]), snap‐trap (−3.5 [IQR −10.8 − 4.75]), and KBS groups (−4.5 [IQR −11.00 − 0.00]). As shown on Fig. 7, the median of the differences between the paired observations was statistically significant at treated sites (Live‐trap: W = 1542.5, *P* = 0.05; Snap‐trap: W = 1215, *P* = 0.05; KBS: W = 1851, *P* < 0.001), indicating a short‐term effect of treatment on in‐house rodent activity. Conversely, in households within the non‐treatment group, there was no difference in the median tile activity score pre‐ and post‐treatment (W = 1698.5, *P* = 0.7).

### Long‐term effect of control

During the post‐treatment trapping sessions at the 12 study sites, 149 rodents were caught inside houses over 835 trap nights. The majority of captures were *M. musculus* (*n* = 105), followed by *R. rattus* (*n* = 43). One *S. murinus* was also captured. A further breakdown of post‐treatment small mammal captures is provided in Table S4b, Supporting Information.

Based on post‐treatment capture data, we did not find evidence that any of the treatments resulted in a decrease in the abundance of rodents in houses when compared to the non‐treatment group. We found no effect of treatment group on capture rate for all rodents combined (χ^2^(3) = 0.53, *P* = 0.9), rats only (χ^2^(3) = 1.96, *P* = 0.58), or mice only (χ^2^(3) = 0.63, *P* = 0.9). Capture rates did vary between villages for all rodents combined (χ^2^(11) = 22.52, *P* = 0.02) and mice only (χ^2^(11) = 22.7, *P* = 0.02).

**Figure 8:**
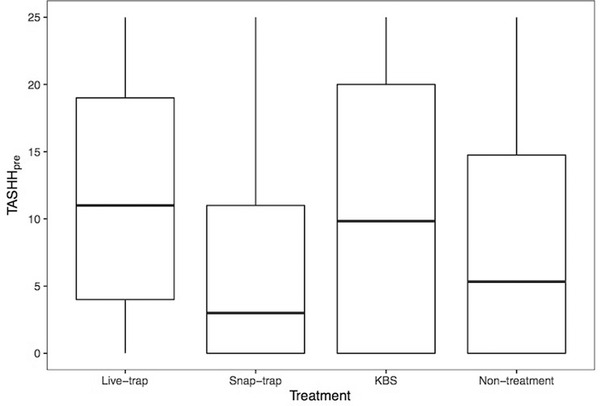
Median, and upper and lower quartiles, of pre‐treatment in‐house tile activity score (TASHH_pre_) recorded during treatment sessions, May–September 2019. Households are grouped by control treatment.

In the analysis to test for a treatment effect on in‐house rodent abundance over the course of the 5 treatment sessions, the variable SEAS provided the best representation of temporal variation in TASHH_pre_. Although in occupied houses the abundance index varied significantly between treatment groups (χ^2^(3) = 11.41, *P* = 0.01), there was no evidence that treatment villages exhibited a different seasonal change in abundance compared to non‐treatment villages as the interaction between treatment and SEAS was not included in the final model (Table S6, Supporting Information). Instead, the effect of treatment was additive, indicating that over the whole treatment period (including the first treatment session) the in‐house rodent abundance index in occupied houses was significantly higher at live‐trap and KBS sites than sites using snap‐traps (Fig. 8). This most likely reflects site to site variation in rodent abundance. Indeed, we also found a negative association between the probability of households scoring zero TASHH_pre_ and TASEXT (χ^2^(1) = 7.69, *P* = 0.005), indicating that the probability of in‐house rodent infestation increased when outdoor rodent abundance was higher. In terms of temporal variation, we found that rodents were recorded in a greater proportion of houses in villages during seasons 2 and 3 (χ^2^(3) = 16.37, *P* < 0.005) compared to season 1, but numbers per house tended to be fewer (χ^2^(3) = 7.88, *P* = 0.02; Table S6, Supporting Information).

**Figure 9:**
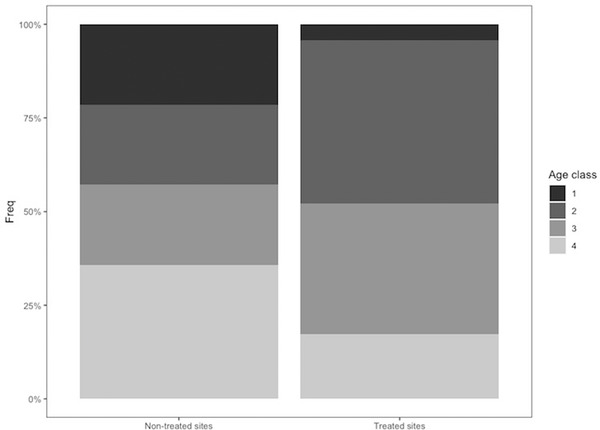
Proportion of *R. rattus* within each age class (1–4) caught during a single post‐treatment trapping session in October 2019, at non‐treated sites and at sites subjected to rodent control treatments.

There is no suggestion that in‐house rats in treated sites showed increased reproduction in the aftermath of this experiment. Indeed, if anything, there was a suggestion that reproduction was lower in these sites during the post‐treatment trapping session. The in‐house rat population in treated sites appeared to have a lower proportion of very young (class 1) and old rats (class 4), and a higher proportion of class 2 and 3 rats than non‐treatment sites (Fig. 9), although this difference in population structure was not significant (Fisher test: *P* = 0.14); while the gestation rate in in‐house rats was 50% (*n* = 6) in non‐treated sites and 17% in treatment sites, but again this difference was non‐significant (Fisher test: *P* = 0.27).

### Diagnostic tests

All spleen (*n* = 923) were tested negative on qPCR/conventional PCR; however, among 794 serum samples tested, 2 were positive on anti F1 IgG antibodies (sera from *R. rattus* caught outdoor in October 2019).

### Fleas

Fifteen (15) *S. fonquerniei* were collected from 44 *R. rattus* trapped inside houses in the post‐treatment session. Of the 11 *R. rattus* caught in villages within the non‐treatment group, none were found to carry *S. fonquerniei*. However, we found no significant difference between treatment groups in the *S. fonquerniei* burden of in‐house *R. rattus* (*T* = 0.46, *P* = 0.9). Instead, we found that house rats carrying *S. fonquerniei* were found in sites where *S. fonquerniei* index of rats caught outside was higher (Mann‐Whitney *U‐*test, *P* = 0.01). The median *S. fonquerniei* burden of rats caught outside was 3.4 at villages where *S. fonquerniei* were also collected from in‐house rats, compared to 0.55 at villages where they were not.

Only 6 *X. cheopis* were collected from *R. rattus* in houses during the post‐treatment trapping session, although a further 5 were collected from 5 *R. rattus* collected outside houses at 3 of the 12 sites. Due to the low numbers of *X. cheopis* on in‐house rats, no statistical analysis was conducted.

During the treatment sessions, a further 52 *S. fonquerniei* and 83 *X. cheopis* were collected from *R. rattus* in houses. As expected, the majority (97.0%) of these came from live‐trap sites. There was clear difference in flea abundance between sites. For example, one live capture site (Ambohimandroso) yielded 98.0% of *S. fonquerniei* collected from rats, but only 7 *X. cheopis*. In contrast, 85.5% of *X. cheopis* were collected from a second live capture site (Andranonomby), from which no *S. fonquerniei* were collected. The third live capture site had a single *S. fonquerniei* and a single *X. cheopis* collected. In addition to the variation between sites in the abundance of these 2 species, a strong effect of house was evident; a single house in site Ambohimandroso provided 62.5% of the infested house rats and 84.3% of the total number of *S. fonquerniei* for that site.

A total of 2608 free fleas were collected with light traps between May and October 2019. Only 5 of these were rodent fleas (4 *S. fonquerniei* and one *X. cheopis)*, whereas the vast majority of sampled fleas were the human flea, *Pulex irritans* (93.8%), as well as *Ctenocephalides felis* (6.0%).

### Outdoor rodents and fleas

Analyses of outdoor rodent abundance revealed significant spatial and temporal variation. TASEXT between May and October showed high levels of rodent activity in the areas surrounding houses, with all sites having at least 1 month where >50% of tiles had rodent tracks in all 25 squares. Change in TASEXT over the 6 months varied between sites, with some showing consistently high activity scores throughout, while others showed some month‐to‐month variation (e.g. ABM), a general increase over time (e.g. TSM), or a decrease over time (e.g. TSR) (Fig. S1, Supporting Information). This variation was reflected in the best model, which included an interaction between village and VISITcat (χ^2^(55) = 304.96, *P* < 0.005).

During the post‐treatment trapping session in all sites, outdoor traps caught 380 *R. rattus* and one *S. murinus* (1233.5 trap nights; Table S4b, Supporting Information). There was significant variation between villages in the abundance of rats (χ ^2^(11) = 66.11, *P* < 0.005).

During the post‐treatment session, 688 *S. fonquerniei* were collected from the 380 *R. rattus*. There was substantial variation between villages in the *S. fonquerniei* flea burden for outdoor rats, ranging from 0.18 to 4.11. The data was best fit by a ZINB model (Table S3, Supporting Information), and there was a positive association between the number of *S. fonquerniei* collected from infested rats and TASEXT during the post‐treatment session (χ ^2^(1) = 4.8, *P* = 0.03).

## DISCUSSION

Fundamental to future plague prevention programs in Madagascar is the control of rodents and their fleas. Our study provides important information to inform the development of future strategies. We found evidence that our live‐trapping regime was more effective than lethal snap‐traps at removing in‐house rodents. However, when implemented over only 5 days per month, controls were insufficient to impact in‐house rodent abundance between months. After 5 months of intermittent control, we found no evidence of increased reproduction among in‐house *R. rattus* populations compared to sites with no control. In fact, there was a suggestion of lower gestation rates and fewer old and very young individuals at treated sites compared to non‐treated sites. Thus, our results are most consistent with treatments being generally effective at removing the resident breeding individuals, but compensatory immigration tends to replace these individuals within the following 4 weeks. Although we found no evidence that in‐house rodents in treatment sites were more likely to be carrying *S. fonquerniei* (the flea usually found on outside rodents) at the end of the experiment, our power to detect such an effect was impacted by significant spatial variation between villages in the outdoor abundance of *S. fonquerniei*, which was the main driver of *S. fonquerniei* presence inside houses. In turn, the abundance of *S. fonquerniei* on outdoor rats was linked to high outdoor rodent abundance, while the probability of rodent infestation in houses was also higher when outdoor rodent abundance was high. Thus, our findings emphasize the connectivity between in‐house and outdoor rat and flea populations at our study site. Further studies should seek to confirm whether similar trends are observed at other sites, particularly in areas of plague risk.

Comparing the effectiveness of live‐traps and snap‐traps, we found the former to be more effective at capturing both *R. rattus* and *M. musculus*. This may be attributed to the use of multiple trap types at live‐trap sites; while BTS traps catch a higher number of *R. rattus*, Sherman traps will catch *M. musculus* and smaller *R. rattus*. Conversely, when using snap‐traps, the lower capture rate of *R. rattus* when mice were present indicated a likely issue of trap saturation, which was not found at live‐trap sites. The capture rate of mice was likewise lower at snap‐trap sites when rats were present, but the relatively low capture rate of rats suggests an impact of rat presence on mouse behavior (Monadjem *et al*. 2011) rather than a trap saturation effect. It is also worth noting that although rodent abundance indices indicated no systematic differences between treatment groups in pre‐treatment rodent abundance, when monthly tracking tile data were analyzed together (with a corresponding increase in statistical power), there was evidence that in‐house rodent abundance was lower at snap‐trap sites than at live‐trap sites. Consequently, although we tried to control for variation in in‐house abundance in the analyses using TASHH_pre_, we cannot completely exclude the possibility that trap success at live‐trap sites may be positively impacted by rodent abundance. Generally, however, our results emphasize that trap type needs to be considered carefully for any future control strategy and highlight the benefit of employing a combination of trap types with different specificity in terms of small mammal captures. Future control strategies will also need to consider whether locally made alternatives to the traps used in this study may be less effective.

In the present study, control treatments were carried out intermittently, over 5 nights each month. At these sites, we found some evidence of a decrease in rodent activity immediately after treatment, but this reduction was not observed in all houses and relatively few houses achieved a zero score for rodent activity. Intensive daily trapping has been shown to reduce rodent populations in rural households; in a plague‐endemic region of Uganda, eradication of in‐house rodents was achieved in a median of 6 days, with households using 4 traps of 2 different types (Eisen *et al*. 2018). However, for some homes, eradication took up to 16 days (Eisen *et al*. 2018). Thus, our results are consistent with those of extended trapping needed to achieve eradication. Our finding that any decrease in rodent activity following treatment did not persist between months was also consistent with findings from the Ugandan study, where, even following apparent eradication from the household, rodent abundance returned to pre‐treatment levels within 8 weeks in 68.9% of households. A study in Namibia, Tanzania, and Swaziland found that sustained reductions in in‐house rodent abundances may be possible if control occurs daily over a long period (12 months) and is organized at community level (Taylor *et al*. 2012). Additional studies are needed to determine the optimal trapping effort needed to achieve rodent control in Malagasy households, while considering the local ecological context and population dynamics of the target species. Importantly, our results highlight that this must include consideration of compensatory immigration from surrounding habitats.

Our comparisons of in‐house rat population structures and gestation rates between treatment and non‐treatment sites (conducted one month after the last treatment cycle), although not significant, point to compensatory immigration rather than compensatory reproduction being primarily responsible for the recovery of in‐house rat populations. Although more studies are needed in this system, this would be consistent with many previous studies of rodent populations (Krebs *et al*. 1978; Sullivan *et al*. 2001, 2003). Compensatory immigration is likely to be a particular problem in Madagascar where *R. rattus* inhabits almost all habitats from villages to forest. This is unlike in East African countries (e.g. Tanzania) and southern Africa (e.g. Swaziland), where *R. rattus* is largely restricted to houses and peri‐domestic settings (Taylor *et al*. 2012). Moreover, in addition to likely limiting the effectiveness of in‐house rodent control, in Malagasy plague foci this immigration from surrounding areas could represent a risk in terms of plague transmission.

Plague transmission in rat populations is thought to be initiated by the seasonal increase in *S. fonquerniei* abundance on rats in agricultural fields and other outdoor habitats, with movement of rats into houses in search of resources resulting in plague transmission to in‐house rat and flea populations and, subsequently, humans (Rahelinirina *et al*. 2010; Andrianaivoarimanana *et al*. 2013). The start of the human plague season in September/October does appear to coincide with the *S. fonquerniei* abundance peak (Klein 1966) and a period of relatively high natural immigration into houses (Rahelinirina *et al*. 2010). However, *S. fonquerniei* numbers are already increasing on outdoor rodent populations by May or June (Rahelinirina *et al*. 2010), and indeed in our study, *S. fonquerniei* were collected from light traps inside 3 houses as early as May and June. Thus, controlling rodents inside houses in the months before the human plague season, with the resultant compensatory immigration from surrounding areas, is not without risk. As this study found that the presence of *S. fonquerniei* inside houses was associated with a high *S. fonquerniei* abundance outside, which in turn was associated with a higher abundance of rodents in outdoor areas, this risk will vary substantially between villages. Moreover, as a single house was responsible for 67% (*n* = 66) of *S. fonquerniei* collected from in‐house rodents, the risk is also likely to fall predominantly in some households. Similarly, we also found that in‐house rat abundance varied greatly between houses within the same village, while rodent abundance in the immediate vicinity of villages (within approximately 80 m) was highly variable between villages. However, as our study was restricted to one season (May–October 2019), we were unable to confirm whether variation in rodent and flea abundances between houses and between villages are consistent year‐on‐year.

A potential benefit of KBS is the simultaneous delivery of rodenticide and insecticide, but whether KBS are an effective tool for flea control requires further investigation (Miarinjara *et al*. 2019). In our study, the abundance of *X. cheopis* (the flea usually found on rodents inside houses) at sites during the experiment period was not sufficient to draw robust conclusions on the impact of control on this flea. The human flea, *P. irritans*, which has been suggested to play a role in the human‐to‐human transmission of *Y. pestis* during outbreaks (Ratovonjato *et al*. 2014), represented the majority of captures in light traps. However, the species is not associated with rodents and so is unlikely to be impacted by insecticides delivered in rodent bait boxes, in addition to the lack of data on their susceptibility to insecticides (Miarinjara *et al*. 2019).

When evaluating the viability of rodent controls as a disease‐prevention strategy, the acceptability of control methods should also be considered. Reporting rodent damage to crops as well as buildings and property, farmers in central and eastern Madagascar identified poisons and snap‐traps as the preferred method of control (Soarimalala *et al*. 2019; Constant *et al*. 2020). This is against general advice in Madagascar to use lethal control within plague foci. However, in our study, participants at KBS sites questioned the efficacy of the slow‐acting rodenticide, and 5 of the 12 households at one of the sites refused to participate in the trial due to the strong smell of the insecticide. Participants also were in favor of live‐traps, due to their perceived effectiveness. One strategy being discussed for rodent control in Madagascar is to reduce rat population density inside houses through continuous community‐level trapping, initiated inside houses during the low plague season and used in combination with an insecticide. However, while insecticide dusting has been shown to be effective in reducing rodent flea burden (Miarinjara *et al*. 2019), their sustained use risks exacerbating the development of insecticide resistance (Miarinjara *et al*. 2017) and is unlikely to provide a long‐term solution to flea control.

The association between outdoor rodent abundance, in‐house infestation, and the presence of *S. fonquerniei* inside houses, highlights the connectivity between households and surrounding areas, and the importance of adopting an ecologically‐based approach to rodent control—using knowledge of the species’ population dynamics, and in particular the density‐ and resource‐dependence of movement and reproduction, to inform control programs. Additional studies are needed to understand what factors drive spatial and temporal variation in the outdoor abundance of rats and *S. fonquerniei*, the risk factors associated with an influx of these rats and fleas into houses both during the human plague season and in the months preceding, and how control strategies will impact on these processes. Finally, we recommend that future rodent control strategies in Madagascar consider the acceptability of proposed methods. Control will likely require persistent, intensive effort on behalf of local communities; factors such as perceived safety and effectiveness, as well as cost and labor requirements, will likely influence the adoption and sustained use of control treatments.

## Supporting information




**Table S1** Schedule of activities carried out at study sites over a 7‐night period
**Table S2** Evaluation of different distributions for GLM(M) analyses
**Table S3** Summary of Generalized Linear Mixed Model (GLMM) analyses of tracking tile, rodent capture, and flea index data
**Table S4a** Breakdown of small mammal and flea capture data, April‐September 2019
**Table S4b** Breakdown of small mammal and flea capture data from post‐treatment trapping session, October 2019
**Table S5** Results of GLMM analyses of variables predicting in‐house rodent, rat and mouse captures in Miantso, Ankazobe District, Madagascar, 2019
**Table S6** Results of GLMM analysis of variables predicting in‐house tile activity scores (TASHH) between April and September 2019, in Miantso, Ankazobe District, Madagascar
**Figure S1** Median, and upper and lower quartiles, of outdoor tile activity score (TASEXT) per village, during each treatment session (visits 1–5) and the post‐treatment session (visit 6)
