# Supplementary material for: Rodent control to fight plague: field assessment of methods based on rat density reduction
Source: Integr Zool. 2021 Mar 30;16(6):868–85. doi: 10.1111/1749-4877.12529 (PMC9291132; doi:10.1111/1749-4877.12529)
Supplement: Supplementary file 1 — Table S1 Schedule of activities carried out at study sites over a 7‐night period Table S2 Evaluation of different distributions for GLM(M) analyses Table S3 Summary of Generalized Linear Mixed Model (GLMM) analyses of tracking tile, rodent capture, and flea index data Table S4a Breakdown of small mammal and flea capture data, April‐September 2019 Table S4b Breakdown of small mammal and flea capture data from post‐treatment trapping session, October 2019 Table S5 Results of GLMM analyses of variables predicting in‐house rodent, rat and mouse captures in Miantso, Ankazobe District, Madagascar, 2019 Table S6 Results of GLMM analysis of variables predicting in‐house tile activity scores (TASHH) between April and September 2019, in Miantso, Ankazobe District, Madagascar Figure S1 Median, and upper and lower quartiles, of outdoor tile activity score (TASEXT) per village, during each treatment session (visits 1–5) and the post‐treatment session (visit 6) [file INZ2-16-868-s001.doc]

**Table S1** Schedule of activities carried out at study sites over a 7-night period. Activities were repeated approximately every four weeks, between May and September 2019.

| **Location** | **Night 1** | **Night 2** | | **Night 3** | | **Night 4** | | **Night 5** | | **Night 6** | | **Night 7** | |
| --- | --- | --- | --- | --- | --- | --- | --- | --- | --- | --- | --- | --- | --- |
| Inside | Tracking tiles | | Control treatment (exc. non-treatment sites) | | | | | | | | | | Tracking tiles |
|  |  | |  | |  | |  | |  | Light trap | | |  |
| Outside | Tracking tiles | |  |  | | | | | | |  | | |

**Table S2** Evaluation of different distributions for GLM(M) analyses. Dependent variables used in each analysis and the visit(s) during which data were collected are given. Pairwise comparisons of the resulting models were based on AIC values, with the differences in AIC values given in the table : a positive change in AIC means the column model is better; a negative change means the row model is better. If the difference in AIC is <2 the simpler model is selected.

| **Dependent variable** |  | **Poisson** | **Negative Binomial (NB)** | **Zero-inflated Poisson (ZIP)** | **Zero-inflated Negative Binomial (ZINB)** |
| --- | --- | --- | --- | --- | --- |
| **Visit 1** | | | | | |
| TASHH_pre_ | Poisson | - | 741.3 | 728.8 | 792.3 |
|  | NB | - | - | -12.5 | 51.0 |
|  | ZIP | - | - | - | 63.4 |
| TASEXT_empty_ | Poisson | - | 957.2 | 574.2 | 959.9 |
|  | NB | - | - | -383.0 | 2.7 |
|  | ZIP | - | - | - | 385.7 |
| **Visits 1-5** | | | | | |
| Rodent captures | Poisson | - | 20.6 | - | - |
|  | NB | - | - | - | - |
|  | ZIP | - | - | - | - |
| *R. rattus* captures | Poisson | - | 10.1 | - | - |
|  | NB | - | - | - | - |
|  | ZIP | - | - | - | - |
| *M. musculus* captures | Poisson | - | 19.4 | - | - |
|  | NB | - | - | - | - |
|  | ZIP | - | - | - | - |
| TASHH_pre_ | Poisson | - | 4113.4 | 3304.7 | 4659.4 |
|  | NB | - | - | -1176.1 | 178.6 |
|  | ZIP | - | - | - | 1216.0 |
| **Visits 1-6** | | | | | |
| TASEXT_empty_ | Poisson | - | 7273.6 | - | - |
|  | NB | - | - | - | - |
|  | ZIP | - | - | - | - |
| **Visit 6** | | | | | |
| Rodent captures | Poisson | - | 2.2 | 2.3 | 0.4 |
|  | NB | - | - | 0.1 | -1.9 |
|  | ZIP | - | - | - | -2.0 |
| *R. rattus* captures | Poisson | - | -0.3 | -2.8 | -4.8 |
|  | NB | - | - | -2.5 | -4.5 |
|  | ZIP | - | - | - | -2.0 |
| *M. musculus* captures | Poisson | - | 5.9 | 6.4 | - |
|  | NB | - | - | 0.5 | - |
|  | ZIP | - | - | - | - |
| *R. rattus* captures (outdoors) | Poisson | - | 2.0 | - | - |
|  | NB | - | - | - | - |
|  | ZIP | - | - | - | - |
| *Synopsyllus fonquerniei* index of outdoor *R. rattus* | Poisson | - | - | 503.03 | 611.32 |
|  | NB | - | - | -100.19 | 8.1 |
|  | ZIP | - | - | - | 108.3 |

**Table S3** Summary of Generalized Linear Mixed Model (GLMM) analyses of tracking tile, rodent capture, and flea index data. For each analysis, the dependent variable and the visit(s) during which data were collected are given, as well as the model distribution used (zero-inflated negative binomial (ZINB), negative binomial (NB) or Poisson), the explanatory variables included in the full model, and the explanatory variables included in the best-selected model. Model parameters were included as additive effects or interactions as indicated by ‘:’. ‘ZI’ indicates variables used in the zero-inflated part of ZINB models. Random effects are indicated by ‘(1|[variable name])’. Abbreviations used: TASHH = household tile activity score ; TASEXT = outdoor tile activity score ; VISIT_cont_ = mission number included as continuous variable ; VISIT_cat_ = mission number included as a categorical variable ; SEAS = a 3-level categorical variable with visit 1, visits 2/3 and visits 4/5 as levels; PR_MM_ / PR_RR_ = presence of M. musculus / R. rattus (a binary yes/no variable).

| **Dependent variable** | **Distribution** | **Explanatory variables included in full model** | **Explanatory variables included in best-selected model** |
| --- | --- | --- | --- |
| Visit 1 | | | |
| TASHH_pre_ | ZINB | TASHH_pre_ ~ village, ZI = ~ village | TASHH_pre_ ~ 1, ZI = ~ village |
|  | ZINB | TASHH_pre_ ~ treatment + (1\|village),  ZI = ~ treatment + (1\|village) | TASHH_pre_ ~ 1 + (1\|village),  ZI = ~ 1 + (1\|village) |
| TASEXT_empty_ | NB | TASEXT_empty_ ~ village | TASEXT_empty_ ~ village |
|  | NB | TASEXT_empty_ ~ treatment + (1\|village) | TASEXT_empty_ ~ 1 + (1\|village) |
| Visits 1-5 | | | |
| Rodent captures | NB | Rodent captures ~ treatment:VISITcont + treatment:TASHH + TASEXT + (1|village/house) | Rodent captures ~ treatment + TASHH + TASEXT + (1|village/house) |
| *R. rattus* captures | NB | *R. rattus* captures ~ treatment:VISIT_cont_ + treatment:TASHH + treatment :PR_MM_ + TASEXT + (1|village/house) | *R. rattus* captures ~ VISIT_cont_ + TASHH + treatment :PR_MM_ + (1|village/house) |
| *M. musculus* captures | NB | *M. musculus* captures ~ treatment:VISIT_cont_ + treatment:TASHH + treatment :PR_RR_ + TASEXT + (1|village/house) | *M. musculus* captures ~ TASHH +  treatment :PR_RR_ + TASEXT + (1|village/house) |
| TASHH_pre_ | ZINB | TASHH_pre_ ~ treatment:SEAS + TASEXT + (1|village/house),  ZI = ~ treatment:SEAS + TASEXT + (1|village/house) | TASHH_pre_ ~ treatment + SEAS (1|village/house), ZI = ~ SEAS + TASEXT + (1|village/house) |
| Visits 1-6 | | | |
| TASEXT_empty_ | NB | TASEXT_empty_ ~ village:VISIT_cat_ + (1|tile.code) | TASEXT_empty_ ~ village:VISIT_cat_ + (1|tile.code) |
| Visit 6 | | | |
| Rodent captures | NB | Rodent captures ~ village | Rodent captures ~ village |
|  | NB | Rodent captures ~ treatment + (1\|village) | Rodent captures ~ 1 + (1\|village) |
| *R. rattus* captures | Poisson | *R. rattus* captures ~ village | *R. rattus* captures ~ 1 |
|  | Poisson | *R. rattus* captures ~ treatment + (1\|village) | *R. rattus* captures ~ 1 + (1\|village) |
| *M. musculus* captures | NB | *M. musculus* captures ~ village | *M. musculus* captures ~ village |
|  | NB | *M. musculus* captures ~ treatment + (1\|village) | *M. musculus* captures ~ 1 + (1\|village) |
| *R. rattus* captures (outdoors) | Poisson | *R. rattus* captures ~ village | *R. rattus* captures ~ village |
| *S. fonquerniei* index of outdoor *R. rattus* | ZINB | *S. fonquerniei* index ~ TASEXT + (1|village),  ZI = ~ TASEXT + (1|village) | *S. fonquerniei* index ~ TASEXT + (1|village),  ZI = ~ 1 + (1|village) |

**Table S4a Breakdown of small mammal and flea capture data, April-September 2019. Free fleas refer to fleas collected in light traps. Rodent fleas refer to fleas collected from rodents caught in live traps (BTS and Sherman (Sh)) and snap-traps.**

|  | **BTS** | **Sh** | **Live traps**^†^ | **Snap-trap** | **KBS** | **Control** |
| --- | --- | --- | --- | --- | --- | --- |
| **Small mammal captures in-house** |  |  |  |  |  |  |
| *M. musculus* | 9 | 162 | 171 | 82 | 3 | - |
| *R. rattus* | 96 | 8 | 104 | 31 | 1 | - |
| *S. murinus* | 0 | 0 | 0 | 1 | 0 | - |
| Total capture | 105 | 170 | 275 | 114 | 4 | - |
| Corrected trap nights | 1159 | 589 | 1748 | 2008 | - | - |
| Trap success | 9.1 | 28.9 | 15.7 | 5.7 | - | - |
| **Free flea collection** |  | | | | | |
| *P. irritans* |  |  | 468 | 514 | 391 | 683 |
| *C. felis* |  |  | 19 | 57 | 26 | 48 |
| *X. cheopis* |  |  | 0 | 1 | 0 | 0 |
| *S. fonquerniei* |  |  | 3 | 0 | 0 | 1 |
| Total free fleas |  |  | 490 | 572 | 417 | 732 |
| House infested |  |  | 76/124 | 73/142 | 71/140 | 92/180 |
| **Rodent flea collection** |  | | | | | |
| *X. cheopis* |  |  | 79 | 4 | - | - |
| *S. fonquerniei* |  |  | 52 | 0 | - | - |
| Total rodent fleas |  |  | 131 | 4 | - | - |
| Flea index |  |  | 0.5 | 0.0 | - | - |
| Total rodents infested |  |  | 25/104 | 3/31 | - | - |

**Table S4b Breakdown of small mammal and flea capture data from post-treatment trapping session, October 2019.**

|  |  |  | | **Live traps**^†^ | | **Snap-trap** | **KBS** | | **Control** |
| --- | --- | --- | --- | --- | --- | --- | --- | --- | --- |
| **Small mammal captures in-house** | | | | | | | | | |
| *M. musculus* |  | |  | 23 | | 26 | 26 | | 30 |
| *R. rattus* |  | |  | 10 | | 7 | 9 | | 17 |
| *S. murinus* |  | |  | 1 | | 0 | 0 | | 0 |
| Total capture |  | |  | 34 | | 33 | 35 | | 47 |
| Corrected trap nights |  | |  | 168 | | 209 | 199 | | 259 |
| Trap success |  | |  | 20.2 | | 15.8 | 17.6 | | 18.1 |
| **Free flea collection** |  | | | |  | | |  | |
| *P. irritans* |  | |  | 7 | | 10 | 83 | | 291 |
| *C. felis* |  | |  | 0 | | 0 | 4 | | 2 |
| *X. cheopis* |  | |  | 0 | | 0 | 0 | | 0 |
| *S. fonquerniei* |  | |  | 0 | | 0 | 0 | | 0 |
| Total free fleas |  | |  | 7 | | 10 | 87 | | 293 |
| House infested |  | |  | 3/23 | | 4/26 | 13/26 | | 23/32 |
| **Rodent flea collection** |  | | | |  | | |  | |
| *X. cheopis* |  | |  | 6 | | 8 | 0 | | 0 |
| *S. fonquerniei* |  | |  | 3 | | 0 | 2 | | 2 |
| *P. irritans* |  | |  | 2 | | 0 | 0 | | 0 |
| Total rodent fleas |  | |  | 11 | | 8 | 2 | | 2 |
| Flea index |  | |  | 0.3 | | 0.2 | 0.1 | | 0.0 |
| **Small mammal captures outdoors** | | | | |  | | |  | |
| *M. musculus* |  | |  | 0 | | 0 | 0 | | 0 |
| *R. rattus* |  | |  | 120 | | 115 | 58 | | 87 |
| *S. murinus* |  | |  | 1 | | 0 | 0 | | 0 |
| Total capture |  | |  | 121 | | 115 | 58 | | 87 |
| Corrected trap nights |  | |  | 297 | | 300.5 | 309 | | 327 |
| Trap success |  | |  | 40.7 | | 38.2 | 18.8 | | 26.6 |
| **Rodent flea collection** |  | |  |  | |  |  | |  |
| *X. cheopis* |  | |  | 1 | | 0 | 3 | | 1 |
| *S. fonquerniei* |  | |  | 228 | | 261 | 118 | | 84 |
| *P. irritans* |  | |  | 3 | | 0 | 0 | | 0 |
| *C. felis* |  | |  | 0 | | 2 | 0 | | 0 |
| Total rodent fleas |  | |  | 232 | | 263 | 121 | | 85 |
| Flea index |  | |  | 1.9 | | 2.3 | 2.1 | | 1.0 |
| ^†^ Live trap = BTS + Sherman | | | | | | | | | |

**Table S5** Results of GLMM analyses of variables predicting in-house rodent, rat and mouse captures in Miantso, Ankazobe District, Madagascar, 2019. Models included a random effect of house nested within village and an offset of the natural log of the number of available traps. Full models included an effect of (VISITcont), in-house tile score (TASHHpre), and outside tile score (TASEXT), and interactions between trap regime and VISITcont and TASHHpre. In species-specific models we included the interaction of trap regime and the presence of the other rodent species (PR_RR_ or PR_MM_). Variables included within the best-selected models are listed in the table below. Model parameters were included as additive effects or interactions as indicated by ‘:’. Negative estimates represent variables associated with a decrease in the number of captures, whilst variables with positive estimates are associated with an increase. Reference level for treatment is live-trap and reference for PR is absence. P-value <0.05*, <0.01**

|  | **Model parameters** | **Parameter estimate** | | | |
| --- | --- | --- | --- | --- | --- |
|  |  | **Estimate** | **SE** | **z value** | **p value** |
| Rodent captures | Intercept | -1.355 | 0.383 | -3.537 | <0.005** |
|  | Treatment (Snap-trap) | -0.789 | 0.275 | -2.83 | <0.005** |
|  | TASHH_pre_ | 0.025 | 0.009 | 2.831 | 0.005** |
|  | TASEXT | -0.038 | 0.018 | -2.106 | 0.035* |
|  | **Random effect** | **Variance** | **SD** |  |  |
|  | House within village | 0.013 | 0.113 | - | - |
|  | Village | 0.071 | 0.266 | - | - |
| Rat captures | Intercept | -2.757 | 0.433 | -6.359 | <0.005** |
|  | Treatment (Snap-trap) | -0.587 | 0.423 | -1.389 | 0.165 |
|  | PR_MM_ (Present) | -0.058 | 0.368 | -0.158 | 0.874 |
|  | Treatment : PR_MM_ | -2.437 | 1.134 | -2.149 | 0.032* |
|  | TASHH_pre_ | 0.028 | 0.016 | 1.726 | 0.084 |
|  | VISIT_cont_ | -0.189 | 0.085 | -2.221 | 0.032* |
|  | **Random effect** | **Variance** | **SD** |  |  |
|  | House within village | 0.568 | 0.754 | - | - |
|  | Village | <0.001 | <0.001 | - | - |
| Mouse captures | Intercept | -1.625 | 0.499 | -3.258 | 0.001** |
|  | Treatment (Snap-trap) | -0.64 | 0.418 | -1.531 | 0.126 |
|  | PR_RR_ (Present) | -0.3 | 0.244 | -1.217 | 0.224 |
|  | Treatment : PR_RR_ | -1.973 | 1.043 | -1.891 | 0.058 |
|  | TASHH_pre_ | 0.034 | 0.012 | 2.826 | 0.005** |
|  | TASEXT | -0.053 | 0.022 | -2.447 | 0.014* |
|  | **Random effect** | **Variance** | **SD** |  |  |
|  | House within village | 0.075 | 0.273 | - | - |
|  | Village | 0.168 | 0.41 | - | - |

**Table S6** Results of GLMM analysis of variables predicting in-house tile activity scores (TASHH) between April and September 2019, in Miantso, Ankazobe District, Madagascar. Models included a random effect of house nested in village and offset of the natural log of the number of tile squares counted. The full model included an interaction effect of treatment and season (SEAS), and an additive effect of outside tile activity score (TASEXT). Variables included within the best-selected models are listed in the table below. In the conditional model, negative estimates represent variables associated with a decrease in TASHH, whilst variables with positive estimates are associated with an increase. In the zero-inflation model, negative estimates indicate a decrease in the probability of 0 scores, and vice versa. P-value <0.05*, <0.01**

| **Model parameters** | **Parameter estimate** | | | |
| --- | --- | --- | --- | --- |
|  | **Estimate** | **SE** | **z value** | **p value** |
| *Conditional model* |  |  |  |  |
| Intercept | -0.331 | 0.134 | -2.464 | 0.014* |
| Treatment (non-tr) | -0.179 | 0.125 | -1.437 | 0.151 |
| Treatment (KBS) | 0.192 | 0.123 | 1.562 | 0.118 |
| Treatment (Snap) | -0.322 | 0.134 | -2.402 | 0.016* |
| SEAS (2) | -0.33 | 0.117 | -2.815 | 0.005** |
| SEAS (3) | -0.354 | 0.116 | -3.043 | <0.005** |
| Random effect | **Variance** | **SD** |  |  |
| House within village | 0.079 | 0.28 | - | - |
| Village | 0.002 | 0.041 | - | - |
| *Zero-inflation model* |  |  |  |  |
| Intercept | -2.487 | 0.57 | -4.369 | <0.005** |
| SEAS (2) | -1.402 | 0.343 | -4.093 | <0.005** |
| SEAS (3) | -1.57 | 0.345 | -4.546 | <0.005** |
| TASEXT | -0.07 | 0.025 | -2.733 | 0.006** |
| Random effect | **Variance** | **SD** |  |  |
| House within village | 1.725 | 1.313 | - | - |
| Village | 0.214 | 0.463 | - | - |


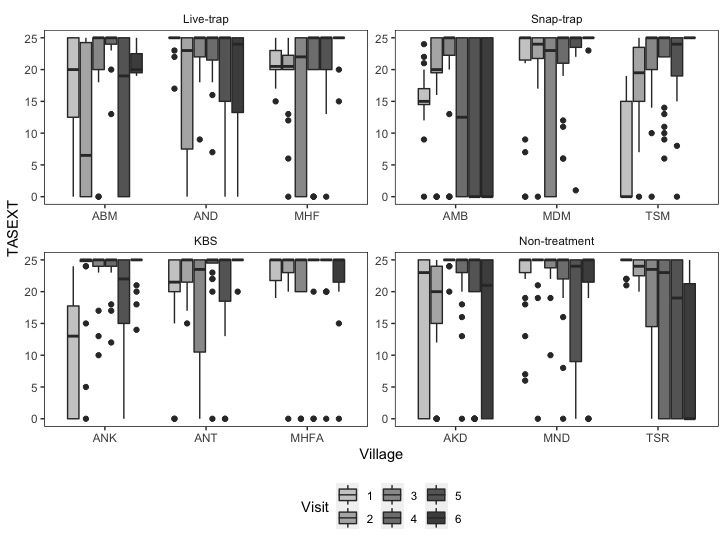


**Figure S1** Median, and upper and lower quartiles, of outdoor tile activity score (TASEXT) per village, during each treatment session (visits 1-5) and the post-treatment session (visit 6). Villages are grouped by treatment (Live-trap: Ambohimandroso (ABM), Andranonomby (AND), Mahafehy (MHF); Snap-trap: Ambohinomehy (AMB), Miadamanjaka (MDM), Tsaramasoandro (TSM); KBS: Anosikely (ANK), Antamboho (ANT), Mahafehy atsimo (MHFA); Non-treatment: Ankadimbazimba (AKD), Mandrosoa (MND), Tsarazoky (TSK))
